# Detection of drug-induced acute respiratory distress syndrome risk signals in FAERS: a real-world pharmacovigilance study

**DOI:** 10.3389/fmed.2026.1820377

**Published:** 2026-06-11

**Authors:** Yunhan Zhao, Feiyang Zhao, Haoxiang Hu, Jianghai He, Yiting Ni, Pingping Jin, Xiao Wang, Zheru Jin, Yiren Hu

**Affiliations:** 1 Department of Neurology, Postgraduate Training Base Alliance of Wenzhou Medical University (Wenzhou People’s Hospital), Wenzhou, China; 2 Wenzhou Third Clinical Institute Affiliated to Wenzhou Medical University, Wenzhou People’s Hospital, Wenzhou, China

**Keywords:** adverse events, ARDS, disproportionality analysis, FAERS, pharmacovigilance

## Abstract

**Background:**

Drug-induced acute respiratory distress syndrome (ARDS) represents an often overlooked yet potentially severe adverse reaction in clinical practice. Existing evidence predominantly stems from isolated case reports, and systematic investigations based on large-scale real-world data remain limited. This study aimed to comprehensively evaluate the risk signals of drug-related ARDS and identify potential high-risk drug classes using the US FDA Adverse Event Reporting System (FAERS).

**Methods:**

We retrieved data from FAERS and extracted ARDS-related reports based on the MedDRA Preferred Terms (PT), followed by deduplication and data cleaning. Four disproportionality analysis methods were used to detect drug signals. LASSO regression and multivariable logistic regression were applied to identify potential independent risk factors. The time interval from drug exposure to ARDS onset was also assessed.

**Results:**

A total of 15,986 drug-related ARDS reports were included, with the highest proportion occurring in middle-aged and older adults, and slightly more cases in males than in females. The five most frequently reported drugs were mycophenolic acid, methotrexate, rituximab, tacrolimus, and amiodarone. Signal detection indicated strong associations for prednisone, mycophenolic acid, amiodarone, and cytarabine. Multivariable analyses further identified 22 drugs significantly associated with ARDS risk. The median time to ARDS onset was 30 days, and approximately 75% of cases occurred within 150 days after treatment initiation.

**Conclusion:**

Using real-world data, this study identified multiple drug classes with significantly elevated ARDS risk, including immunosuppressants, antineoplastic agents, glucocorticoids, cardiovascular drugs, and nonsteroidal anti-inflammatory drugs (NSAIDs). These findings provide important evidence for clinical monitoring of high-risk medications.

## 1 Introduction

Acute respiratory distress syndrome (ARDS) is a clinical emergency characterized by acute hypoxemia, bilateral pulmonary infiltrates, and inflammatory lung injury, often accompanied by diffuse pulmonary inflammation and associated with high mortality rates (1). ARDS has diverse and complex etiologies, including common triggers such as infection, trauma, and aspiration. Among these causes, drug-induced ARDS represents a distinct subtype that is frequently overlooked in clinical practice. Although its underlying pathophysiology has not been fully elucidated, current evidence suggests that multiple biological pathways may be involved, including hypersensitivity reactions, direct pulmonary toxicity, immune-mediated injury, and drug-related endothelial dysfunction (2–4).

Although case reports have suggested that various drugs—including antineoplastic agents, anti-infective drugs, and immunomodulators—may induce or exacerbate ARDS, most of the evidence is derived from sporadic individual cases, and a systematic analytical framework is lacking (5–7). For example, trimethoprim-sulfamethoxazole (TMP–SMX), antiarrhythmic agents, nonsteroidal anti-inflammatory drugs (NSAIDs), rifampicin, and mesalazine have all been reported to be associated with ARDS onset (2, 5–8). yet the strength of these observations remains limited. In addition, the diagnosis of drug-induced ARDS requires strict exclusion of alternative causes and relies heavily on temporal association, making clinical recognition challenging and often delaying risk assessment (9). Furthermore, existing studies predominantly focus on single drugs or small patient cohorts, and systematic risk evaluation based on large-scale real-world data is still lacking (10, 11). As a result, the true risk distribution, high-risk drug classes, and susceptible patient populations for drug-induced ARDS remain unclear and insufficiently quantified.

Real-world pharmacovigilance data offer a valuable opportunity to address these evidence gaps. The US FDA Adverse Event Reporting System (FAERS), one of the largest adverse event databases worldwide, has substantial advantages in identifying rare, delayed, or unexpected adverse reactions (12, 13). Signal detection based on FAERS enables systematic evaluation of potential associations between drugs and ARDS in large populations and allows the identification of safety signals that may be difficult to capture in conventional clinical studies. Therefore, this study utilized the FAERS database to systematically identify drug signals associated with ARDS, evaluate the risk intensity across drug classes, and explore potential patient-related risk factors, with the aim of providing robust evidence to support improved pharmacovigilance strategies, promote early clinical recognition, and facilitate individualized treatment.

## 2 Methods

### 2.1 Data source

This study consists of three main steps: (1) data collection, (2) drug information standardization and organization, and (3) statistical analysis. A retrospective pharmacovigilance analysis was conducted using the FAERS database. The FAERS database contains post-market surveillance data for all approved drugs and therapeutic biologics (14). Since 2004, it has continuously collected adverse event (AE) reports from healthcare professionals, patients, and drug manufacturers, and the database is regularly updated. FAERS includes detailed information such as patient demographics, drug information, indications, AE occurrence times, and related clinical outcomes (15). As FAERS is a publicly available anonymized database, this study did not require approval from an institutional review board or patient informed consent. We utilized the MedDRA (Medical Dictionary for Regulatory Activities) 26.0 version Preferred Terms (PT) for data retrieval, collecting all AE reports from the first quarter of 2004 to the fourth quarter of 2024. Specifically, our goal was to identify drug-related AE reports associated with “drug-induced ARDS.”

### 2.2 Statistical analysis

**Figure 1:**
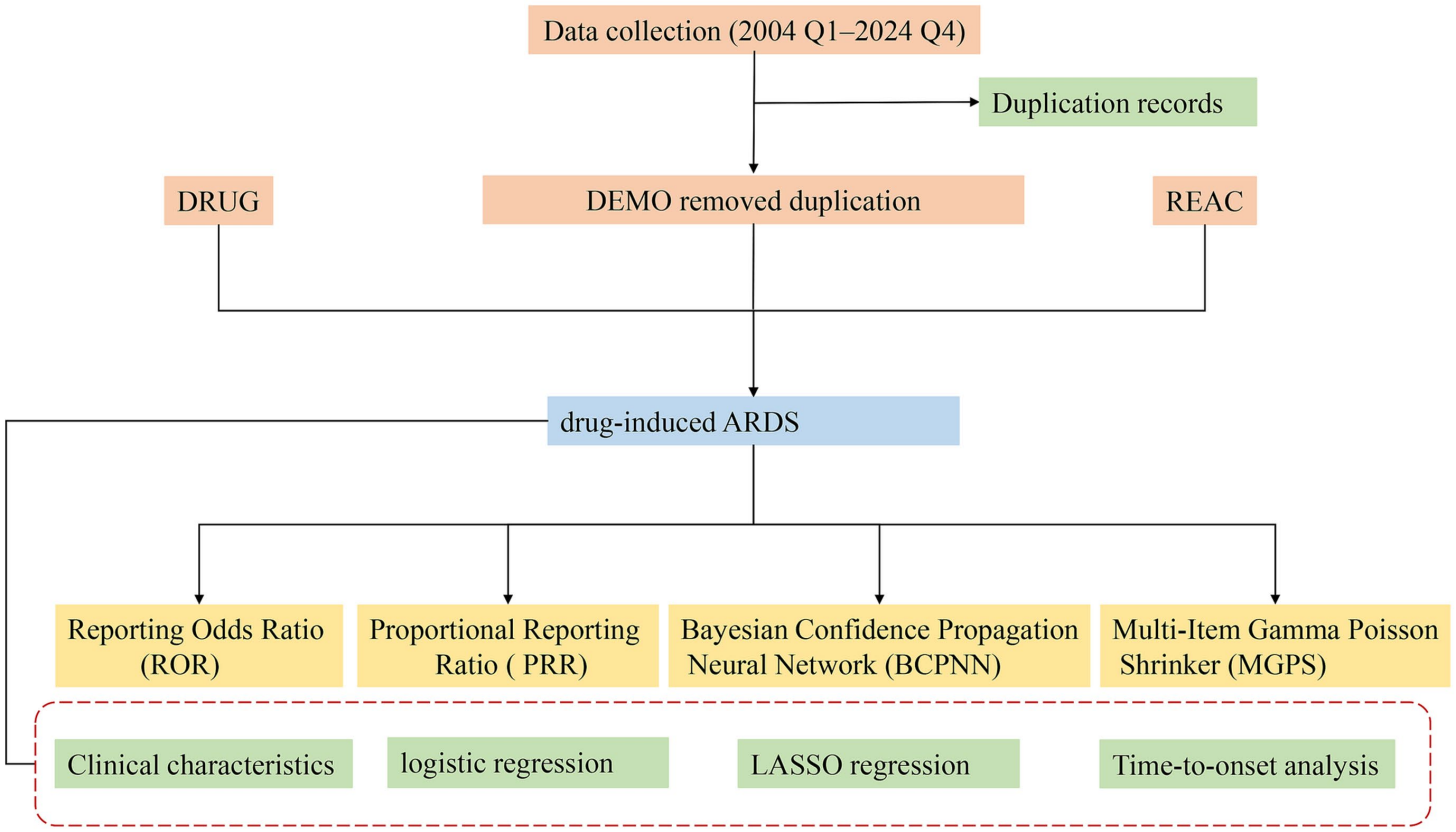
The flowchart of this study.

Data cleaning was performed according to the official FAERS database procedures (16). For statistical analysis, we first used descriptive statistics to summarize clinical characteristics of drug-induced ARDS patients, including age, sex, reporting year, reporter type, and country. To identify potential drug safety signals, we performed disproportionality analysis, a pharmacovigilance data-mining approach used to detect drug–adverse event pairs that are reported more frequently than expected relative to the background reporting frequency in a spontaneous reporting database. We then employed four disproportionality analysis methods to quantitatively assess the strength of the association between drugs and ARDS, including Reporting Odds Ratio (ROR), Proportional Reporting Ratio (PRR), Empirical Bayes Geometric Mean (EBGM), and Information Component (IC). ROR identifies potential signals by calculating the ratio of the probability of an AE report for a specific drug to the probability of reports for other drugs. A higher ROR value indicates a stronger likelihood of an association between the drug and the event (17). PRR evaluates the ratio of reports for a specific event for a drug to reports for all other drugs. A positive ROR signal was defined when the lower bound of the 95% confidence interval exceeded 1 (ROR > 1) and the number of related reports was at least 3. A potential PRR signal was defined as PRR > 2, *χ*^2^ > 4, and at least 3 related reports. In general, higher ROR or PRR values indicate a stronger disproportional reporting association between the drug and ARDS (18). Both EBGM and IC are Bayesian methods that compare the observed number of reports to the expected number based on background rates to identify signals exceeding expectations. EBGM > 1 and IC > 0 suggest a potential association, and the lower bound of their 95% confidence interval (EB05 and IC025) was used to assess signal robustness (19, 20). Detailed formulas and criteria for identifying positive signals are provided in Supplementary Table 1. To assess the statistical significance of drug–ARDS associations, Fisher’s exact test was performed for each drug. Because multiple comparisons were involved, *p* values were adjusted using the Bonferroni correction method to reduce the risk of false-positive findings. The adjusted p values were subsequently used for significance evaluation and volcano plot visualization. To further refine the risk factors, we conducted chi-square tests and univariate logistic regression analysis for drugs with positive signals. Drugs that met specific criteria (ROR > 1, report count > 100, *p* < 0.01) were then included in the Least Absolute Shrinkage and Selection Operator (LASSO) regression model to identify variables most strongly associated with ARDS occurrence. LASSO is a penalized regression method that shrinks less informative coefficients toward zero, thereby facilitating variable selection and reducing overfitting. Finally, a multivariable logistic regression model was built to control for potential confounding factors, such as age and sex, and to determine independent risk factors for drug-induced ARDS. Additionally, we analyzed the time-to-onset of ARDS after drug exposure. The overall workflow of the study is shown in Figure 1. All data processing and statistical analyses were performed using R software (version 4.4.1).

## 3 Results

### 3.1 Baseline characteristics of drug-induced ARDS

A total of 15,986 drug-related ARDS reports were included in this study, covering the period from the first quarter of 2004 to the fourth quarter of 2024. The gender distribution showed that 42.8% (7,843 cases) were female, 46.7% (8,558 cases) were male, and 10.5% (1,926 cases) had missing gender information. In terms of age distribution, the largest proportion of reports occurred in patients aged 41–64 years (4,807, 30.1%), followed by those aged ≥65 years (4,061, 25.4%), 19–40 years (2,400, 15.0%), and ≤18 years (1,411, 8.8%). Age information was unknown in 3,307 reports (20.7%). Geographically, the highest number of reports came from the United States (37.9%, 6,053 cases), followed by Germany (10.8%, 1,726 cases) and Japan (5.8%, 929 cases). Regarding report sources, healthcare professionals submitted the highest proportion of reports, accounting for 16.4%. The temporal distribution of reports across the study period is presented in Table 1. Reports were observed throughout 2004Q1 to 2024Q4, with relatively higher proportions in the most recent years, particularly 2022, 2023, and 2024.

**Table 1:** Baseline characteristics of patients with drug-associated ARDS.

Characteristics (*N* = 15,986)	Reports, *n* (%)
Sex	
Female	6,843 (42.8%)
Male	7,464 (46.7%)
Unknown	1,679 (10.5%)
Age	
≤18	1,411 (8.8%)
19–40	2,400 (15.0%)
41–64	4,807 (30.1%)
≥65	4,061 (25.4%)
Unknown	3,307 (20.7%)
Occupation of the reporter	
Health professional	2,623 (16.4%)
Lawyer	78 (0.5%)
Consumer	1,341 (8.4%)
Other	10,797 (67.5%)
Unknown	1,147 (7.2%)
Reported countries	
United States	6,053 (37.9%)
France	1,726 (10.8%)
Japan	929 (5.8%)
Unknown	803 (5.0%)
Other	6,475 (40.5%)
Reported years	
2004	479 (3.0%)
2005	492 (3.1%)
2006	509 (3.2%)
2007	470 (2.9%)
2008	489 (3.1%)
2009	522 (3.3%)
2010	592 (3.7%)
2011	593 (3.7%)
2012	618 (3.9%)
2013	654 (4.1%)
2014	638 (4.0%)
2015	646 (4.0%)
2016	681 (4.3%)
2017	810 (5.1%)
2018	903 (5.6%)
2019	977 (6.1%)
2020	1,445 (9.0%)
2021	1,215 (7.6%)
2022	1,075 (6.7%)
2023	1,019 (6.4%)
2024	1,159 (7.3%)

### 3.2 Descriptive analysis

**Figure 2:**
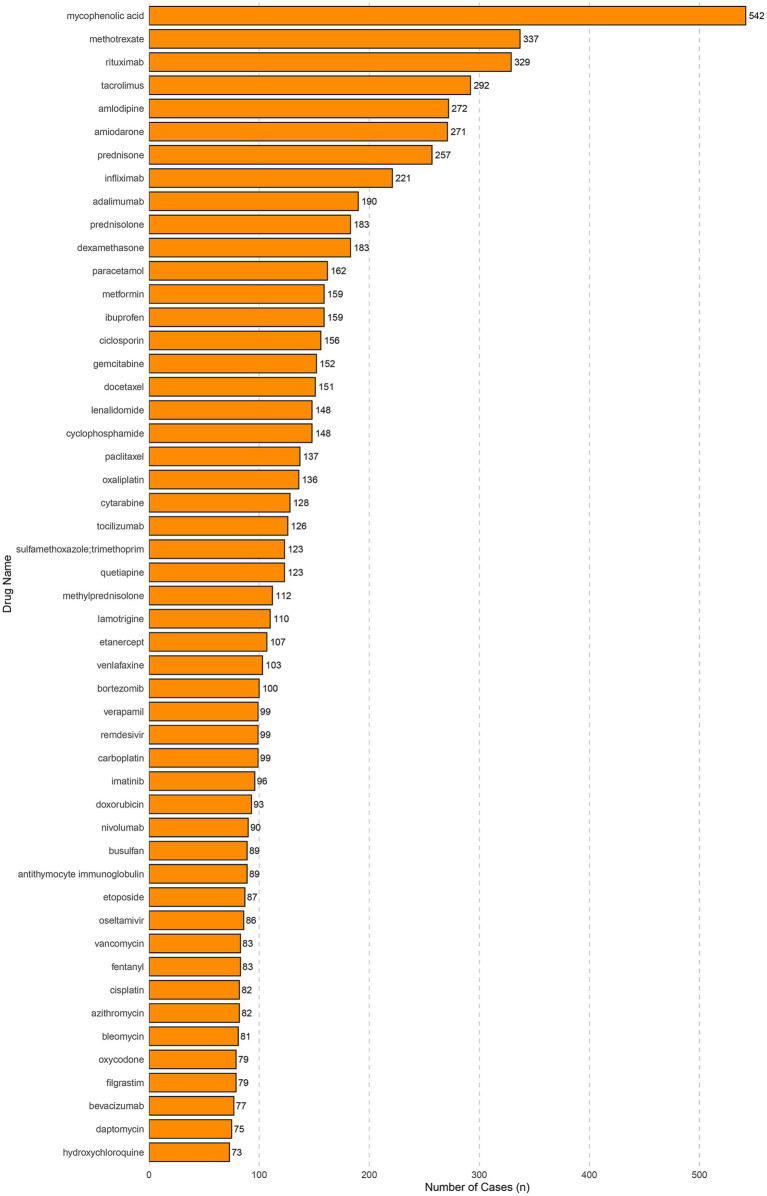
Top 50 drugs associated with ARDS based on case counts reported in the FAERS database. Horizontal bar chart illustrating the top 50 drugs most frequently associated with ARDS in the FAERS database from 2004Q1 to 2024Q4. Each bar represents the number of reported ARDS cases linked to a specific drug.

Based on the FAERS database, we further summarized the top 50 drugs most frequently associated with drug-induced ARDS (Table 2). Mycophenolic acid was the most frequently reported drug (542 cases, 3.39%), followed by methotrexate (337 cases, 2.11%), rituximab (329 cases, 2.06%), tacrolimus (292 cases, 1.83%), and amiodarone (271 cases, 1.70%) (Figure 2). Among these 50 drugs, antineoplastic agents were the most common (15 drugs), followed by corticosteroids (4 drugs), antimicrobial agents (4 drugs), antiarrhythmic agents (2 drugs), antiviral drugs (2 drugs), as well as antihypertensive agent, immunosuppressants, biologics, and antipsychotic medications. Notably, a review of product labeling showed that amiodarone, rituximab, tacrolimus, cyclophosphamide, docetaxel, cytarabine, filgrastim, nivolumab, and bortezomib explicitly mentioned ARDS in their prescribing information, whereas most of the remaining drugs did not provide a clear ARDS warning.

**Table 2:** Disproportionality analysis of drugs associated with ARDS in the FAERS database.

Drug	N	ROR (95% CI)	PRR (*χ*^2^)	EBGM (EB05)	IC (IC25)
Mycophenolic acid	542	11 (10.09–11.99)	10.91 (4716.98)	10.57 (9.84)	3.4 (3.28)
Methotrexate	337	3.07 (2.76–3.42)	3.07 (460.14)	3.02 (2.76)	1.6 (1.44)
Rituximab	329	3.76 (3.37–4.19)	3.75 (650.53)	3.69 (3.37)	1.89 (1.72)
Tacrolimus	292	5.81 (5.17–6.52)	5.79 (1135.99)	5.7 (5.17)	2.51 (2.34)
Amlodipine	272	6.49 (5.76–7.32)	6.46 (1235.11)	6.37 (5.76)	2.67 (2.49)
Amiodarone	271	16.28 (14.42–18.37)	16.07 (3767.98)	15.81 (14.29)	3.98 (3.81)
Prednisone	257	8.66 (7.66–9.8)	8.61 (1701.68)	8.49 (7.65)	3.08 (2.9)
Infliximab	221	1.47 (1.29–1.68)	1.47 (32.65)	1.46 (1.31)	0.55 (0.35)
Adalimumab	190	0.35 (0.31–0.41)	0.35 (223.46)	0.36 (0.32)	−1.47 (−1.68)
Dexamethasone	183	5.14 (4.44–5.95)	5.12 (600.76)	5.08 (4.49)	2.34 (2.13)
Prednisolone	183	6.46 (5.58–7.47)	6.43 (829.9)	6.37 (5.63)	2.67 (2.46)
Paracetamol	162	2.07 (1.77–2.42)	2.07 (88.34)	2.06 (1.81)	1.04 (0.81)
Ibuprofen	159	2.35 (2.01–2.75)	2.35 (121.82)	2.33 (2.05)	1.22 (0.99)
Metformin	159	2.66 (2.28–3.11)	2.66 (163)	2.64 (2.32)	1.4 (1.17)
Ciclosporin	156	3.68 (3.14–4.31)	3.67 (300.8)	3.65 (3.2)	1.87 (1.64)
Gemcitabine	152	6.81 (5.81–8)	6.78 (742.67)	6.73 (5.88)	2.75 (2.51)
Docetaxel	151	3.47 (2.96–4.08)	3.47 (262.79)	3.44 (3.01)	1.78 (1.55)
Cyclophosphamide	148	5.84 (4.97–6.87)	5.82 (585.79)	5.78 (5.04)	2.53 (2.29)
Lenalidomide	148	0.49 (0.42–0.58)	0.49 (78.05)	0.49 (0.43)	−1.02 (−1.25)
Paclitaxel	137	4.5 (3.8–5.32)	4.48 (367.89)	4.45 (3.87)	2.15 (1.91)
Oxaliplatin	136	5.05 (4.26–5.98)	5.03 (436.04)	5 (4.34)	2.32 (2.07)
Cytarabine	128	12.69 (10.65–15.11)	12.56 (1352.2)	12.47 (10.77)	3.64 (3.38)
Tocilizumab	126	2.59 (2.18–3.09)	2.59 (122.03)	2.58 (2.22)	1.37 (1.11)
Quetiapine	123	1.85 (1.55–2.21)	1.85 (47.97)	1.85 (1.59)	0.88 (0.62)
Sulfamethoxazole; Trimethoprim	123	12.12 (10.14–14.48)	12 (1231.98)	11.92 (10.27)	3.57 (3.31)
Methylprednisolone	112	5.23 (4.34–6.3)	5.21 (379.15)	5.18 (4.44)	2.37 (2.1)
Lamotrigine	110	2.47 (2.05–2.98)	2.47 (95.6)	2.46 (2.1)	1.3 (1.02)
Etanercept	107	0.24 (0.2–0.29)	0.24 (258.15)	0.24 (0.21)	−2.04 (−2.31)
Venlafaxine	103	2.88 (2.37–3.49)	2.87 (125.12)	2.86 (2.43)	1.52 (1.23)
Bortezomib	100	3.63 (2.98–4.41)	3.62 (188.38)	3.6 (3.05)	1.85 (1.56)
Carboplatin	99	2.66 (2.18–3.24)	2.66 (101.63)	2.65 (2.24)	1.4 (1.11)
Remdesivir	99	14.52 (11.91–17.72)	14.36 (1223.79)	14.28 (12.09)	3.84 (3.54)
Verapamil	99	16.69 (13.68–20.37)	16.47 (1431.1)	16.38 (13.86)	4.03 (3.74)
Imatinib	96	1.99 (1.63–2.43)	1.99 (46.98)	1.98 (1.68)	0.99 (0.69)
Doxorubicin	93	3.35 (2.73–4.11)	3.35 (152.16)	3.33 (2.81)	1.74 (1.44)
Nivolumab	90	1.64 (1.33–2.02)	1.64 (22.28)	1.63 (1.37)	0.71 (0.41)
Antithymocyte Immunoglobulin	89	18.58 (15.06–22.92)	18.31 (1449.23)	18.21 (15.28)	4.19 (3.88)
Busulfan	89	17.06 (13.83–21.04)	16.83 (1318.9)	16.74 (14.05)	4.07 (3.76)
Etoposide	87	8.62 (6.98–10.66)	8.57 (578.99)	8.53 (7.14)	3.09 (2.78)
Oseltamivir	86	6.74 (5.45–8.33)	6.7 (415.51)	6.67 (5.59)	2.74 (2.43)
Fentanyl	83	1.31 (1.05–1.62)	1.31 (5.97)	1.31 (1.09)	0.38 (0.07)
Vancomycin	83	4.55 (3.66–5.64)	4.53 (227.42)	4.51 (3.77)	2.17 (1.86)
Azithromycin	82	3.96 (3.19–4.92)	3.95 (179.93)	3.94 (3.28)	1.98 (1.66)
Cisplatin	82	5.18 (4.17–6.44)	5.16 (274.07)	5.14 (4.29)	2.36 (2.04)
Bleomycin	81	64.78 (51.77–81.07)	61.43 (4794.96)	61.12 (50.67)	5.93 (5.61)
Filgrastim	79	7.6 (6.09–9.49)	7.56 (447.76)	7.53 (6.25)	2.91 (2.59)
Oxycodone	79	0.61 (0.49–0.77)	0.61 (19)	0.62 (0.51)	−0.7 (−1.02)
Bevacizumab	77	1.3 (1.04–1.63)	1.3 (5.4)	1.3 (1.08)	0.38 (0.05)
Daptomycin	75	10.02 (7.98–12.58)	9.94 (600.97)	9.9 (8.18)	3.31 (2.97)
Hydroxychloroquine	73	5.31 (4.21–6.68)	5.29 (252.73)	5.27 (4.34)	2.4 (2.06)

ROR, reporting odds ratio; PRR, proportional reporting ratio; EBGM, empirical Bayes geometric mean; EB05, lower 95% confidence bound of the EBGM; IC, information component based on the Bayesian Confidence Propagation Neural Network method; IC25, lower 95% confidence bound of the IC.

### 3.3 Drugs associated with ARDS

**Figure 3:**
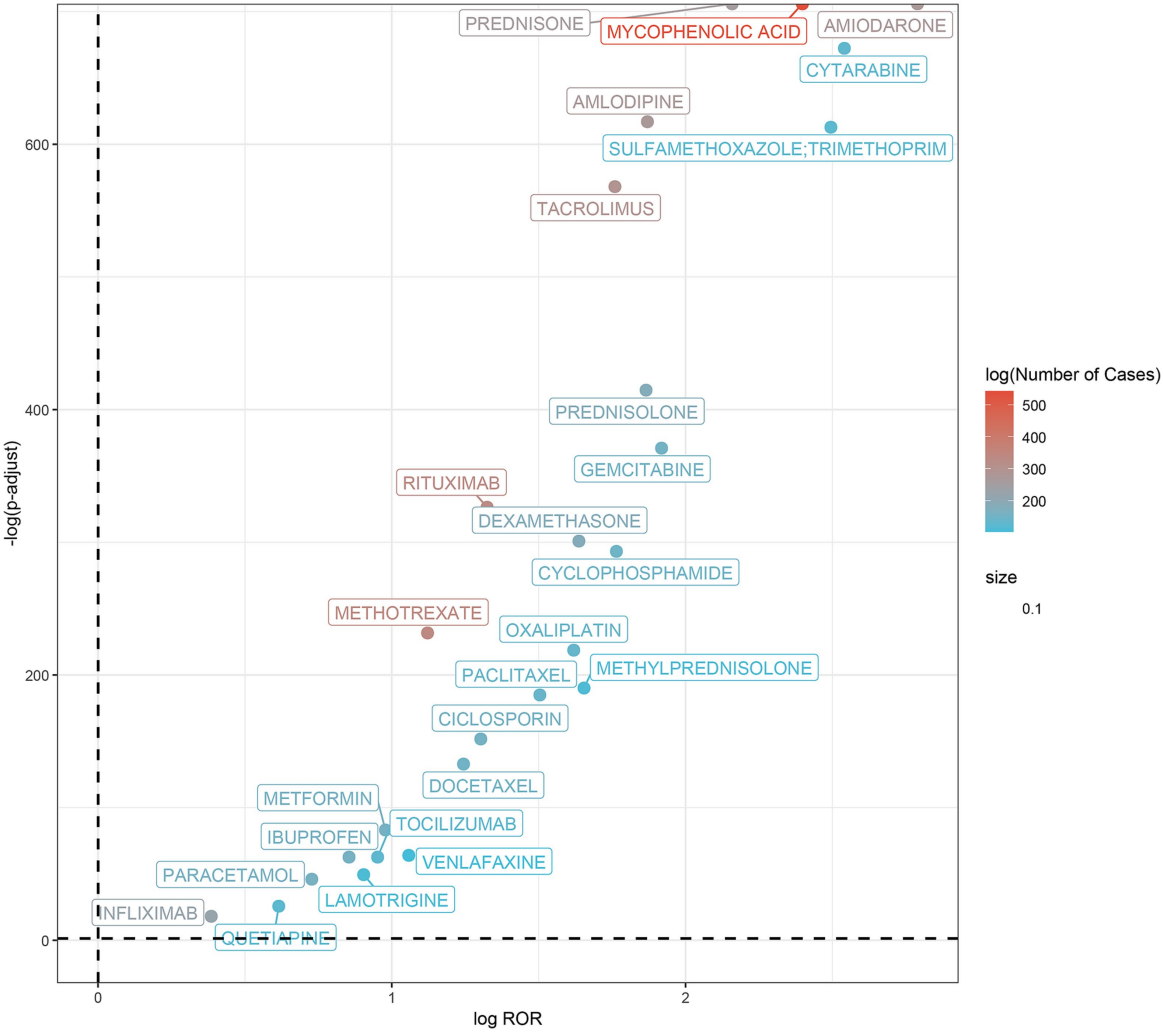
Volcano plot of disproportionality signals for drugs associated with ARDS in the FAERS database. This volcano plot illustrates the disproportionality signals of drugs associated with ARDS based on FAERS data. The *x*-axis represents the log-transformed reporting odds ratio (log ROR), and the *y*-axis represents the negative log-transformed Bonferroni-adjusted *p*-values (−log₁₀(*p*-adjust)). Each point corresponds to a drug, with color intensity indicating the log number of reported cases and point size reflecting signal magnitude. Drugs located in the upper-right quadrant exhibit both strong effect size and high statistical significance, representing the most prominent safety signals. Dashed lines indicate conventional thresholds for signal detection.

Table 3 lists the drugs that met the criteria of ROR (95% CI) > 1, n > 100, and *p* < 0.01 in univariate analysis, and the volcano plot was used to analyze the association between ARDS and suspected drugs (Figure 3). In this plot, the X-axis represents the logarithmically transformed reporting odds ratio (log ROR), which reflects the relative strength of the disproportionality signal for each drug, whereas the Y-axis represents the negative logarithm of the Bonferroni-adjusted *p*-value [−log10(p-adjust)] derived from Fisher’s exact test, which indicates the statistical significance of the association. The color of the points indicates the logarithm of the number of reports, with red indicating higher report frequency. Therefore, drugs in the upper-right quadrant show stronger and more significant signal intensity. The results indicate that drugs highly associated with ARDS include prednisone (ROR = 8.66, 95% CI = 7.66–9.8, PRR = 10.91, *χ*^2^ = 4716.98, EBGM = 10.57, IC = 3.4), mycophenolic acid (ROR = 11.00, 95% CI = 10.09–11.99, PRR = 10.91, *χ*^2^ = 4716.98, EBGM = 10.57, IC = 3.4), amiodarone (ROR = 16.28, 95% CI = 14.42–18.37, PRR = 16.07, *χ*^2^ = 3767.98, EBGM = 15.81, IC = 3.98), and cytarabine (ROR = 12.69, 95% CI = 10.65–15.11, PRR = 12.56, *χ*^2^ = 1352.2, EBGM = 12.47, IC = 3.64). These drugs were located in the significant region of the volcano plot, indicating strong signals.

**Table 3:** Top drugs associated with ARDS based on disproportionality analysis in the FAERS database.

Drug	Case reports (*n*)	ROR (95% CI)	PRR (*χ*^2^)	EBGM (EB05)	IC (IC25)
Mycophenolic Acid	542	11 (10.09–11.99)	10.91 (4716.98)	10.57 (9.84)	3.4 (3.28)
Methotrexate	337	3.07 (2.76–3.42)	3.07 (460.14)	3.02 (2.76)	1.6 (1.44)
Rituximab	329	3.76 (3.37–4.19)	3.75 (650.53)	3.69 (3.37)	1.89 (1.72)
Tacrolimus	292	5.81 (5.17–6.52)	5.79 (1135.99)	5.7 (5.17)	2.51 (2.34)
Amlodipine	272	6.49 (5.76–7.32)	6.46 (1235.11)	6.37 (5.76)	2.67 (2.49)
Amiodarone	271	16.28 (14.42–18.37)	16.07 (3767.98)	15.81 (14.29)	3.98 (3.81)
Prednisone	257	8.66 (7.66–9.8)	8.61 (1701.68)	8.49 (7.65)	3.08 (2.9)
Infliximab	221	1.47 (1.29–1.68)	1.47 (32.65)	1.46 (1.31)	0.55 (0.35)
Dexamethasone	183	5.14 (4.44–5.95)	5.12 (600.76)	5.08 (4.49)	2.34 (2.13)
Prednisolone	183	6.46 (5.58–7.47)	6.43 (829.9)	6.37 (5.63)	2.67 (2.46)
Paracetamol	162	2.07 (1.77–2.42)	2.07 (88.34)	2.06 (1.81)	1.04 (0.81)
Ibuprofen	159	2.35 (2.01–2.75)	2.35 (121.82)	2.33 (2.05)	1.22 (0.99)
Metformin	159	2.66 (2.28–3.11)	2.66 (163)	2.64 (2.32)	1.4 (1.17)
Ciclosporin	156	3.68 (3.14–4.31)	3.67 (300.8)	3.65 (3.2)	1.87 (1.64)
Gemcitabine	152	6.81 (5.81–8)	6.78 (742.67)	6.73 (5.88)	2.75 (2.51)
Docetaxel	151	3.47 (2.96–4.08)	3.47 (262.79)	3.44 (3.01)	1.78 (1.55)
Cyclophosphamide	148	5.84 (4.97–6.87)	5.82 (585.79)	5.78 (5.04)	2.53 (2.29)
Paclitaxel	137	4.5 (3.8–5.32)	4.48 (367.89)	4.45 (3.87)	2.15 (1.91)
Oxaliplatin	136	5.05 (4.26–5.98)	5.03 (436.04)	5 (4.34)	2.32 (2.07)
Cytarabine	128	12.69 (10.65–15.11)	12.56 (1352.2)	12.47 (10.77)	3.64 (3.38)
Tocilizumab	126	2.59 (2.18–3.09)	2.59 (122.03)	2.58 (2.22)	1.37 (1.11)
Quetiapine	123	1.85 (1.55–2.21)	1.85 (47.97)	1.85 (1.59)	0.88 (0.62)
Sulfamethoxazole; Trimethoprim	123	12.12 (10.14–14.48)	12 (1231.98)	11.92 (10.27)	3.57 (3.31)
Methylprednisolone	112	5.23 (4.34–6.3)	5.21 (379.15)	5.18 (4.44)	2.37 (2.1)
Lamotrigine	110	2.47 (2.05–2.98)	2.47 (95.6)	2.46 (2.1)	1.3 (1.02)
Venlafaxine	103	2.88 (2.37–3.49)	2.87 (125.12)	2.86 (2.43)	1.52 (1.23)

ROR, reporting odds ratio; PRR, proportional reporting ratio; EBGM, empirical Bayes geometric mean; EB05, lower 95% confidence bound of the EBGM; IC, information component based on the Bayesian Confidence Propagation Neural Network method; IC25, lower 95% confidence bound of the IC.

### 3.4 Risk factors for drug-related ARDS

**Figure 4:**
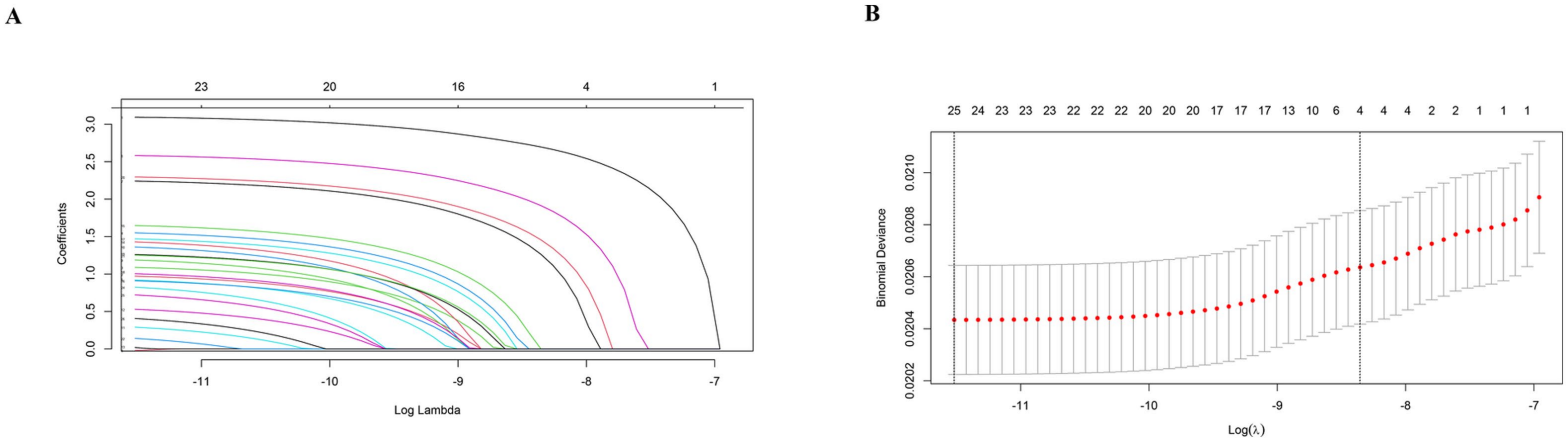
LASSO regression analysis for identifying drug-related risk factors of ARDS. **(A)** Illustrates how each drug’s regression coefficient varies with log(*λ*). As λ increases (moving left to right), coefficients shrink progressively toward zero, reflecting the penalization effect of LASSO and enabling variable selection. **(B)** Displays the binomial deviance across a range of λ values, with error bars representing cross-validation variability. The left dashed line corresponds to the λ that minimizes deviance (λmin), while the right dashed line indicates the λ yielding the most parsimonious model within one standard error (λ1SE). The numbers above the curve denote the count of non-zero coefficients retained at each λ level.

**Figure 5:**
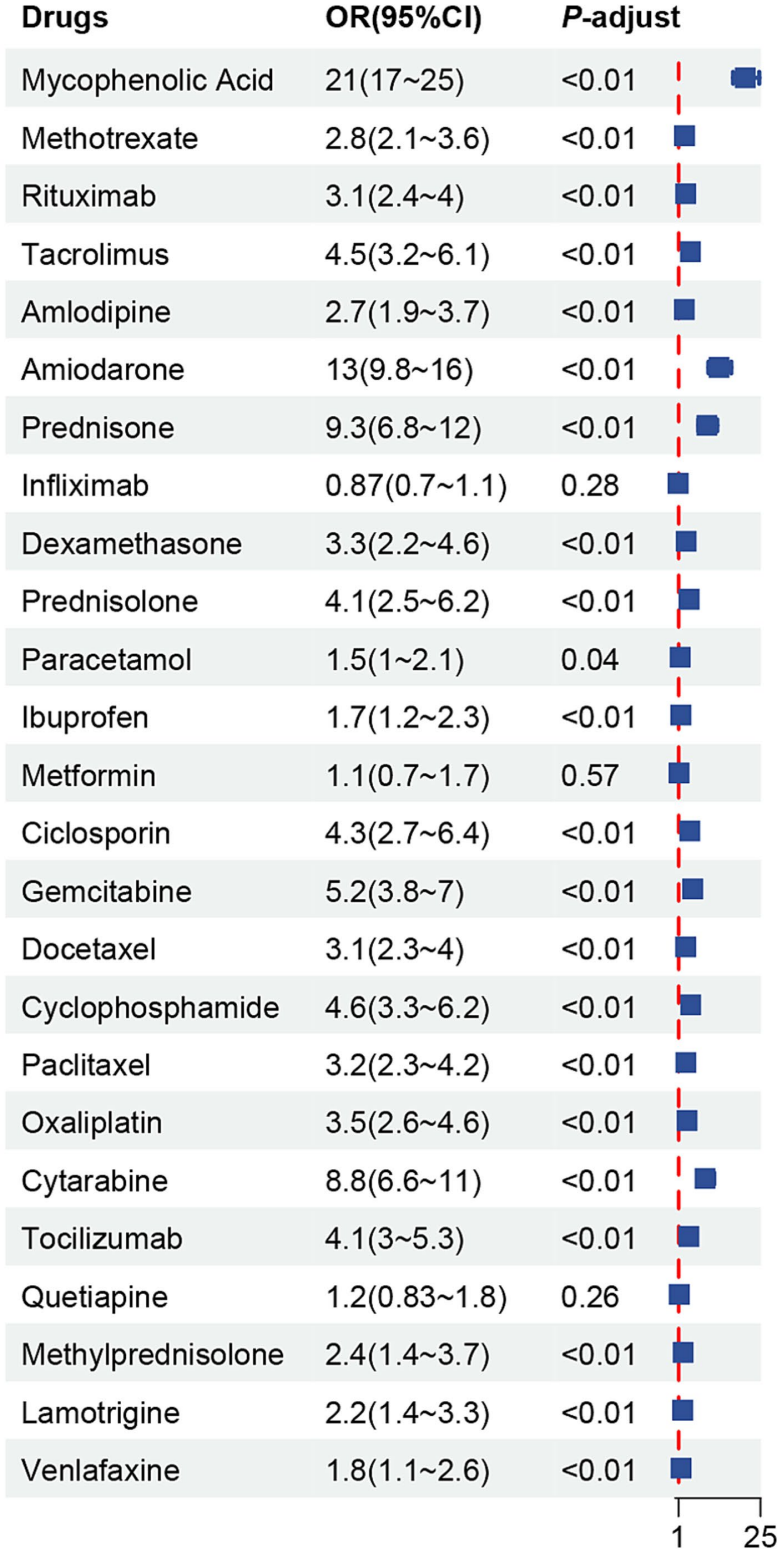
Forest plot of adjusted odds ratios for drugs associated with ARDS in the FAERS database. Forest plot illustrating the adjusted odds ratios (OR) with 95% confidence intervals for drugs associated with ARDS based on multivariable logistic regression analysis.

To further explore potential risk factors for drug-induced ARDS, we performed LASSO regression on drugs that met the criteria of ROR (95% CI) > 1, n > 100, and *p* < 0.01 in univariate analysis, resulting in 25 candidate drugs (Figure 4). Considering the potential confounding effects of age and sex, we conducted multivariate logistic regression analysis on these drugs. The results revealed that 22 drugs were significantly associated with the risk of drug-induced ARDS (Figure 5). Drugs identified in the multivariate regression as significantly related to ARDS risk included: 6 immunosuppressants (mycophenolic acid, methotrexate, rituximab, tacrolimus, cyclosporine, and cyclophosphamide), 4 corticosteroids (prednisone, dexamethasone, prednisolone, and methylprednisolone), 5 antineoplastic agents (gemcitabine, docetaxel, paclitaxel, oxaliplatin, and cytarabine), 2 biologics/immunomodulators (infliximab and tocilizumab), 2 cardiovascular drugs (amlodipine and amiodarone), 2 NSAIDs (acetaminophen and ibuprofen), 3 neuropsychiatric drugs (quetiapine, venlafaxine, and lamotrigine); and 1 antidiabetic drug (metformin). Notably, in the multivariate analysis, prednisone (OR = 4.1, 95% CI = 2.5–6.2, *p* < 0.01), mycophenolic acid (OR = 21, 95% CI = 17–25, *p* < 0.01), amiodarone (OR = 13, 95% CI = 9.8–16, *p* < 0.01), and cytarabine (OR = 8.8, 95% CI = 6.6–11, *p* < 0.01) remained significantly associated with ARDS, suggesting that these drugs have stable and strong associations with ARDS.

### 3.5 Time interval between drug use and ARDS onset

**Figure 6:**
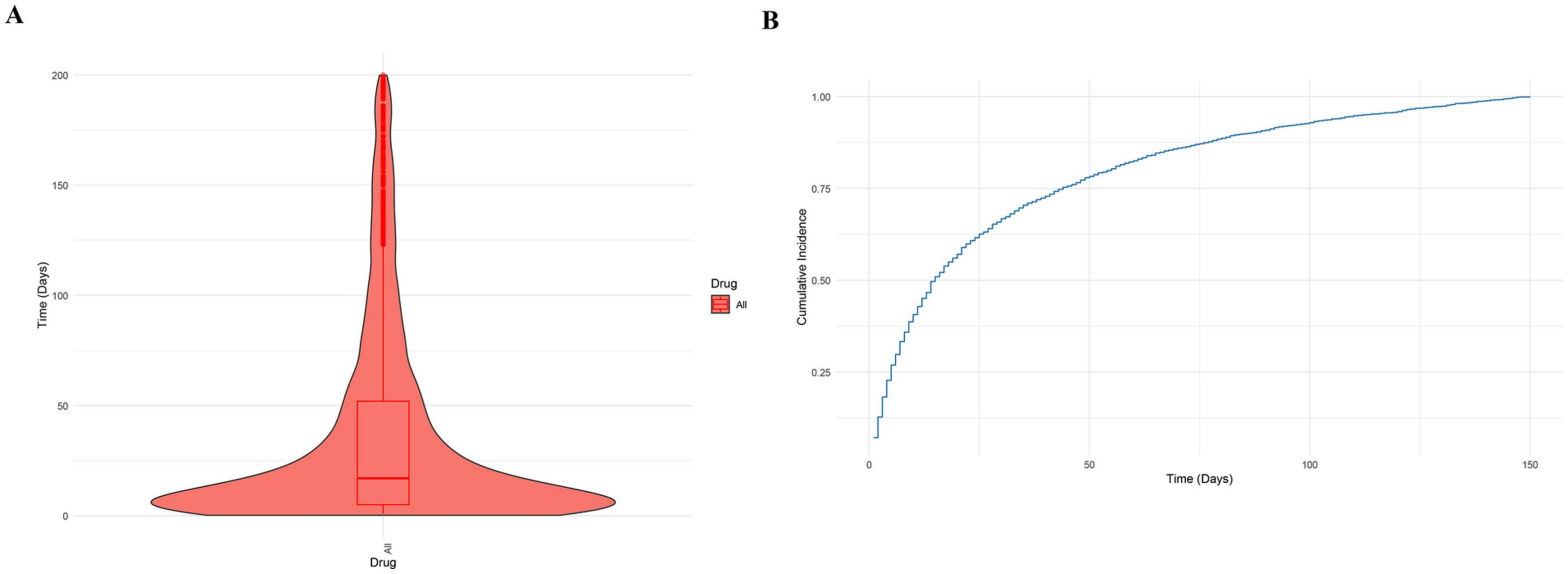
Time-to-onset distribution of ARDS. **(A)** Displays the overall distribution of time-to-onset for drug-associated ARDS cases. **(B)** Illustrates the cumulative proportion of ARDS cases over time.

Additionally, we evaluated the time interval between drug exposure and the onset of ARDS. The results showed that the median time to onset of drug-related ARDS was 30 days (IQR 7–102 days), with approximately 75% of cases occurring within 150 days after drug initiation (Figure 6).

## 4 Discussion

ARDS is a clinical syndrome triggered by various etiologies, characterized by acute respiratory failure and diffuse alveolar injury. Its pathogenesis is highly complex and can be driven by multiple factors, including infection, trauma, and inhalation of toxic substances. Drug-induced ARDS, as a rare but clinically important subtype of ARDS, has attracted increasing attention in recent years. However, its precise prevalence remains unclear because current evidence is derived mainly from case reports, case series, and pharmacovigilance databases rather than population-based epidemiological studies. In addition, because its presentation often overlaps with that of underlying diseases or concomitant infections, establishing a causal relationship between a specific drug and ARDS remains challenging (21).

This study analyzed 18,327 reports related to ARDS, and the results showed that the median age of drug-related ARDS patients was 58 years, with the highest proportion in the 45–65 age group (42.3%). This age distribution aligns with previous studies. Xu et al. analyzed the risk factors for ARDS and found a median age of 56.5 years in patients (22). A retrospective cohort study of critically ill trauma patients based on the US trauma registry (2007–2016) also found that the peak incidence of ARDS occurred in adults aged 35–64 years (23). On the other hand, different studies report significant variation in the median age of ARDS onset, with Riviello et al. reporting a median age of 37 years (24). El-Solh et al. proposed that underlying diseases and comorbidities may alter the age window for ARDS onset (25). Sánchez-Montalvá et al. further observed that non-Hispanic ARDS patients had a higher Charlson Comorbidity Index after age adjustment, suggesting that genetic susceptibility and environmental exposure differences may influence the age of onset (26). Overall, drug-induced ARDS is primarily observed in middle-aged and elderly populations, likely related to the complexity of comorbidities, higher medication use, and cumulative exposure in these individuals. Instead, it is more likely to reflect the combined effects of multiple factors, including the demographic structure of the reported population, patterns of drug exposure, underlying disease profiles, and potential genetic background (27–29). At the same time, older patients are more likely to have multiple comorbidities, polypharmacy, and altered drug metabolism and clearance, all of which may further influence the age distribution of drug-related adverse events (27). Current evidence suggests that genetic susceptibility to ARDS may involve several pathophysiological processes, including inflammatory responses, vascular permeability, immune regulation, and heterogeneity in host responses (28, 29). Previous reviews and GWAS studies have reported that multiple genetic loci are associated with the risk or clinical outcomes of ARDS, including PPFIA1, SELPLG, FLT1, BORCS5, and DUSP16 (28–31). In addition, inflammation-related pathways such as IL-6/IL-6R and NF-κB have also been suggested to participate in the development and progression of ARDS (28, 32, 33). Polymorphisms in the HBD-1 gene have also been reported to affect genetic susceptibility to ARDS and its prognosis (34). From the perspective of population differences, ARDS-related genetic signals are not entirely consistent across different populations. Moreover, age itself is an important risk factor for ARDS, as the overall incidence of ARDS generally increases with age, and elderly patients are at higher risk of severe disease and poor outcomes (35). Notably, the high proportion of reports from the United States may not only be related to differences in drug availability and reporting habits, but also to the fact that FAERS is a U. S.-based spontaneous reporting database, which may inherently introduce geographic reporting bias. In addition, the increase in reports observed between 2022 and 2024 may reflect multiple contributing factors, including strengthened pharmacovigilance, increased awareness of severe respiratory adverse events after the COVID-19 pandemic, and possible changes in medication use patterns (36, 37). This result highlights the importance of continuous monitoring of drug-induced severe pulmonary injury in the context of public health events.

In terms of gender distribution, the reports of drug-related ARDS were 6,843 cases in females and 7,464 cases in males, with a slightly higher proportion in males. This finding is consistent with Zhao et al.’s study on sepsis-induced ARDS, which found a significantly higher incidence in males (61% vs. 39%), and a higher in-hospital mortality rate in males (*p* = 0.009). Male gender was identified as an independent risk factor for ARDS (aOR = 1.493) (38). Mechanistically, Swart et al. and Yin et al. suggested that females may have a stronger innate immune response early in sepsis, while males are more prone to an imbalanced inflammatory response and organ injury, which partly explains the higher incidence of ARDS in males (39, 40). However, since the FAERS data lacks detailed exposure information, whether gender constitutes an independent risk factor for drug-related ARDS still needs further validation in prospective studies controlling for confounding factors.

This study identified several drug signals significantly associated with ARDS, including immunosuppressants (mycophenolic acid, methotrexate, rituximab, tacrolimus, ciclosporin, tocilizumab), chemotherapy drugs (gemcitabine, paclitaxel, cyclophosphamide, docetaxel, oxaliplatin, cytarabine), NSAIDs (ibuprofen, fluconazole), cardiovascular drugs (amlodipine, amiodarone), corticosteroids (prednisone, dexamethasone, prednisolone, methylprednisolone), and antipsychotics (lamotrigine, venlafaxine). These findings provide important clues for clinical identification of high-risk drugs.

Although specific studies on the mechanisms of immunosuppressants inducing ARDS at the drug level are still lacking, substantial evidence supports a close relationship between immunosuppressive states and ARDS. Sanchez et al. and Michal et al. indicated that immunosuppressants significantly increase the risk of pulmonary infections, particularly with opportunistic pathogens. Infection can directly cause epithelial and endothelial damage in the alveoli, triggering an inflammatory cascade that ultimately leads to ARDS (41, 42). Michal et al. further proposed that immunosuppressants may inhibit Treg function, allowing inflammation to dominate and exacerbating alveolar damage. Frans et al. suggested that inappropriate immune suppression, either due to improper dosage or timing, can lead to impaired pathogen clearance and trigger compensatory inflammation (43). Moreover, under immunosuppressive conditions, innate immune cells may be aberrantly activated through the NF-κB pathway, releasing large amounts of inflammatory cytokines that directly disrupt the alveolar-capillary barrier (44, 45). Overall, the key mechanisms by which immunosuppressants cause ARDS involve both “increased susceptibility to infections” and “imbalanced inflammatory regulation,” ultimately leading to barrier disruption, pulmonary edema, and respiratory failure. Enhancing regulatory immune cell function or employing barrier-protective strategies may hold potential therapeutic value.

Currently, the molecular mechanisms underlying chemotherapy drug-induced ARDS remain unclear, but existing research suggests that multiple pathways may be involved. Yu et al. indicated that drug-induced ARDS is commonly observed after exposure to antineoplastic agents, and one potential mechanism is allergic reactions (46). For example, IgE-mediated type I hypersensitivity reactions can induce mast cells to release inflammatory mediators, causing capillary leakage and non-cardiogenic pulmonary edema. Marcella Prete et al. discussed the potential role of cytokine storms in the development of ARDS, suggesting that chemotherapy drugs may excessively activate immune cells, releasing pro-inflammatory cytokines such as IL-6 and TNF-*α*, driving systemic inflammation and damaging the alveolar-capillary barrier (30, 47).

This study also suggests that ibuprofen and fluconazole may be associated with ARDS. Existing studies have shown that fluconazole exerts its antifungal effects primarily by inhibiting fungal ergosterol synthesis. Its known adverse effects are mainly concentrated in hepatotoxicity, renal dysfunction, and cardiotoxicity, such as QT interval prolongation (48–50). ARDS is not considered a typical adverse reaction of fluconazole. On the other hand, fluconazole is an inhibitor of CYP2C9 and CYP3A4, and may therefore indirectly increase the toxic exposure of concomitant medications by affecting their metabolic clearance. In addition, the possibility of idiosyncratic hypersensitivity reactions or immune-mediated lung injury cannot be completely excluded (51, 52). Currently, clinical evidence of NSAIDs directly causing ARDS remains limited, and most studies focus on their toxicity to the gastrointestinal tract, kidneys, etc. (53). The significant heterogeneity of ARDS itself may also contribute to differences in drug responses. Therefore, the causal relationship between NSAIDs and ARDS still requires further validation in larger studies and mechanistic research (54). The ARDS associated with amiodarone is usually mediated by multiple pathological processes, including direct cytotoxicity, mitochondrial dysfunction, immune-inflammatory responses, and oxidative stress (55, 56). Among them, the deposition of phospholipid complexes in the lungs is considered one of the core mechanisms (57). Chao Yang et al. demonstrated that amiodarone could induce oxidative stress and apoptosis through the activation of the AMPK pathway, providing potential targets for its pulmonary toxicity prevention (58). In contrast, the mechanism of amlodipine-induced ARDS is more unclear. Larsen H Welsh et al. reported a case of ARDS in a teenager caused by calcium channel blocker overdose, suggesting that inflammatory responses and increased vascular permeability following drug toxicity may contribute to the onset (59, 60). Moreover, amlodipine, as a calcium channel blocker, may inhibit hypoxic pulmonary vasoconstriction and affect oxygenation, especially exacerbating hypoxemia during lung ventilation (61). Overall, existing evidence supports that amlodipine-induced ARDS may be related to systemic responses following drug toxicity, abnormal pulmonary circulation regulation, and inflammatory/oxidative stress processes, but further empirical research is needed.

Corticosteroids show a significant dose-dependent association with ARDS. Long-term or high-dose use may increase the risk of secondary infections by inhibiting neutrophil chemotaxis and macrophage function, and infection is one of the most important triggers of ARDS. According to existing studies, in the context of ARDS treatment, “long-term use” usually refers to treatment regimens lasting more than 7 days or longer (62). “High dose,” however, is more appropriately understood as a dose exceeding the conventional recommended regimen or one that may result in significant systemic adverse effects, rather than a single fixed absolute value. For example, in COVID-19-related ARDS, dexamethasone 6 mg/day for approximately 10 days is a commonly used regimen (63). In clinical practice, the choice of glucocorticoid type, dose, and duration should be individualized according to the patient’s specific condition, the etiology and severity of ARDS, and the presence of potential comorbidities (62, 63). Karolina A Zielińska et al. found that dexamethasone may exacerbate lung injury due to immune suppression in a malaria-related ARDS model (64). Moreover, corticosteroid resistance (GC resistance) may prevent effective suppression of inflammation. Barnes et al. suggested that GC resistance is an obstacle in treating inflammatory diseases such as COPD and ARDS, indicating that long-term exposure may lead to receptor dysfunction and uncontrolled inflammation. Therefore, corticosteroids may exert anti-inflammatory protective effects at certain stages but could exacerbate lung injury through immune suppression and oxidative stress in the early stages of inflammation or when GC resistance is present (65–67). Although some glucocorticoids showed higher ROR values, this does not necessarily indicate stronger direct pulmonary toxicity. These findings may be influenced by indication bias, since glucocorticoids are often used in patients with severe inflammation, critical illness, or a high baseline risk of respiratory failure. Confounding by disease severity may also increase the apparent association with ARDS. In addition, differences in anti-inflammatory potency, mineralocorticoid activity, route of administration, treatment timing, and clinical use scenarios among glucocorticoids may contribute to differences in signal strength. Therefore, these ROR results should be interpreted cautiously and should not be directly equated with causality.

Currently, there are no direct reports on the association between lamotrigine and ARDS. However, the development of ARDS due to venlafaxine may result from a combination of mechanisms, including immune abnormalities, oxidative stress, adrenergic signaling dysregulation, and direct cellular damage. Although pulmonary toxicity related to venlafaxine is rare, reports suggest it may cause interstitial lung disease and eosinophilic pneumonia, implying potential direct or indirect toxicity to lung tissue. Considering the pathological roles of oxidative stress, endoplasmic reticulum stress, and apoptosis in ALI/ARDS research, it is possible that venlafaxine may increase the risk of ARDS through similar pathways (68–71). Its exact mechanism requires further confirmation through basic experimental and large-scale clinical studies. Clinically, early respiratory symptoms should be closely monitored, and timely drug withdrawal interventions should be performed.

This study also evaluated the time interval between drug exposure and ARDS onset. The median time to onset of drug-related ARDS was 30 days (IQR 7–102 days), with approximately 75% of cases occurring within 150 days after the initiation of the drug. This result suggests that the latent period of drug-induced ARDS is variable, presenting as an acute or subacute process. Although some patients develop respiratory injury early after treatment initiation, a considerable proportion appear to develop ARDS months later. This heterogeneity in time to onset may reflect differences in the mechanisms of drug-induced lung injury, which have been proposed to include direct cytotoxic injury, immune-mediated injury, and metabolite-related injury (4, 72–74). For example, chemotherapy drugs and immunosuppressants often drive lung injury through cumulative toxicity or immune abnormalities over time rather than immediately. Additionally, approximately 25% of cases developed ARDS after 151 days, suggesting that some drug toxicities are progressive and require long-term follow-up and dynamic monitoring First, some forms of drug-related lung injury may not represent an immediate reaction following short-term exposure, but rather may be associated with long-term or cumulative exposure, with pulmonary damage gradually progressing over time and eventually manifesting as ARDS. Second, polypharmacy and drug–drug interactions may alter drug exposure levels and thereby prolong the time to toxicity onset (75); Previous studies have shown that potential drug–drug interactions are relatively common among patients with ARDS. For example, fluconazole is a strong inhibitor of CYP2C9 and CYP3A4, and when used concomitantly with drugs metabolized by these enzymes, it may prolong the half-life of co-administered agents and increase their accumulation (48, 52). In addition, diagnostic delay and reporting delay are also important factors that must be considered in FAERS-based studies. The diagnosis of ARDS requires integration of clinical and imaging criteria, as well as exclusion of other common causes such as infection and cardiogenic pulmonary edema. In patients with atypical presentations, a relatively indolent disease course, or persistent respiratory symptoms in the post-COVID period, the recognition and reporting of drug-related lung injury may both be delayed (76, 77). Therefore, we interpret cases occurring after 150 days as the result of multiple interacting factors, rather than attributing them to a single mechanism.

This study also has some limitations. First, FAERS relies on voluntary reporting, which may introduce reporting bias and lead to overestimation or underestimation of some drug signals. Second, the reports often lack key clinical details (e.g., underlying diseases, laboratory indicators, treatment history), making it difficult to fully control for confounding factors, thus limiting causal inference. Third, since the majority of FAERS cases come from the United States, the findings may not fully represent drug use patterns and adverse event spectra in other countries or regions, potentially affecting external validity. Finally, FAERS lacks a control group and systematic follow-up, meaning that it can only suggest associations, not causality. Therefore, this study is more suitable for signal detection and hypothesis generation, and the conclusions should be further verified through prospective clinical studies and mechanistic experiments.

## 5 Conclusion

This study used real-world data from the FAERS database to systematically identify potential safety signals of drug-induced ARDS. The analysis revealed that several drug classes, including immunosuppressants, antineoplastic agents, corticosteroids, cardiovascular drugs, and NSAIDs, were significantly associated with the risk of ARDS. Among these drugs, mycophenolic acid, amiodarone, prednisone, and cytarabine showed the strongest associations, indicating that their clinical use requires heightened vigilance.

## Data Availability

The original contributions presented in the study are included in the article/Supplementary material, further inquiries can be directed to the corresponding author/s.
