# Supplementary material for: Detection of drug-induced acute respiratory distress syndrome risk signals in FAERS: a real-world pharmacovigilance study
Source: Front Med (Lausanne). 2026 Jun 11;13:1820377. doi: 10.3389/fmed.2026.1820377 (PMC13294336; doi:10.3389/fmed.2026.1820377)
Supplement: Supplementary file 1 [file Data_Sheet_1.pdf]

Supplementary Material

Table 1 2 × 2 fourfold table of disproportionality method.

	Drug of interest	Other drugs	Total
AE of interest	a	b	a + b
Other AEs	c	d	c + d
Total	a + c	b + d	N = a + b + c + d

Abbreviations: AEs, adverse events.

Table 2 ROR, PRR, BCPNN, and EBGM methods, formulas, and thresholds.

Method	Formula	Threshold
ROR	$\mathrm{ROR} = \frac{a/c}{b/d}$<br>$\mathrm{SE(lnROR)} = \sqrt{\frac{1}{a} + \frac{1}{b} + \frac{1}{c} + \frac{1}{d}}$<br>$95\%\mathrm{CI} = e^{\ln(\mathrm{ROR}) \pm 1.96se}$	$a \geq 3$<br>ROR≥ 3<br>95%CI(lower limit) > 1
PRR	$\mathrm{PRR} = \frac{a/(a+b)}{c/c+d}$<br>$\mathrm{SE(lnPRR)} = \frac{1}{a} - \frac{1}{a+b} + \frac{1}{c} - \frac{1}{c+d}$<br>$95\%\mathrm{CI} = e^{\ln(\mathrm{PRR}) \pm 1.96se}$	$a \geq 3$<br>ROR≥ 2<br>95%CI(lower limit) > 1
BCPNN	$\mathrm{IC} = \log_2 \frac{p(x,y)}{p(x)p(\gamma)} = \log_2 \frac{a(a+b+c+d)}{(a+b)(a+c)}$<br>$\mathrm{E(IC)} = \log_2 \frac{(a+\gamma^{11})(a+b+c+d+a)(a+b+c+d+\beta)}{(a+b+c+d+\gamma)(a+b+\alpha 1)(a+c+\beta 1)}$<br>$\mathrm{V(IC)} = \frac{1}{(\ln 2)^2}[\frac{(a+b+c+d)-a+\gamma-\gamma^{11}}{(a+\gamma^{11})} + \frac{(a+b+c+d)-(a+b)-\alpha 1}{(a+b+\alpha 1)(a+b+c+d+\alpha)}$<br>$+ \frac{(a+b+c+d+a)-(a+c)+\beta-\beta 1}{(a+b+\beta 1)(1+a+b+c+d+\beta)}$<br>$\gamma = \gamma^{11} \frac{(a+b+c+d+a)(a+b+c+d+\beta)}{(a+b+\alpha 1)(a+c+\beta 1)}$<br>$\mathrm{IC} - 2\mathrm{SD} = \mathrm{E(IC)} - 2\sqrt{\mathrm{V(IC)}}$	IC025>0

EBGM	$$\mathrm{EBGM} = \frac{a(a+b+c+d)}{(a+b)(a+c)}$$ $$\mathrm{SE}(\ln \mathrm{EBGM}) = \sqrt{\frac{1}{a}+\frac{1}{b}+\frac{1}{c}+\frac{1}{d}}$$ $$95\%\mathrm{CI} = e^{\ln(\mathrm{EBGM}) \pm 1.96\mathrm{se}}$$	EBGM05> 2

Abbreviations: 95% CI, 95% confidence interval; N, the number of reports; χ^2, chi-squared; IC, information component; IC025, the lower limit of 95% CI of the IC; EBGM, empirical Bayesian geometric mean; EBGM05, the lower limit of 95% CI of EBGM
